# WHAT ABOUT THE HANDS? EXAMINING OLDER ADULTS’ ATTITUDES, PERCEPTIONS, AND CLINICAL INTERACTIONS OF HAND FUNCTION

**DOI:** 10.1093/geroni/igae098.1210

**Published:** 2024-12-31

**Authors:** Rachel Logue Cook, Susan Brown

**Affiliations:** University of Michigan, Ann Arbor, Michigan, United States; University of Michigan, Ann Arbor, Michigan, United States

## Abstract

Age-related hand impairments are common yet receive little attention, thus often go undiagnosed until they have become severe. While it has been suggested that attitudes, perceptions, and clinical interactions play an important role in seeking care for physical limitations, the degree to which this extends to hand impairments is unknown. We developed an online survey which asked older adults (≥65) about their perceptions of hand function compared to other health areas, clinical interactions related to hand function, and barriers to accessing hand-related care. Five hundred and ninety-five older adults (mean age: 71.8 ± 5.5y) completed the survey. While 85% of respondents agreed that hand function is important for their daily life, only 40% expressed concern about losing function and only 2% ranked hand function as most important. Responses also indicated that hand function is rarely discussed (21%) or measured (52%) in the primary care setting. In addition, 34% of respondents said they have considered seeking care for a limitation but chose not to because they did not think their function was poor enough (33%), thought function would improve on its own (17%), or thought losing function is part of getting older (16%). Our results demonstrate that, although older adults understand the importance of hand function for completing activities, it is not prioritized by individuals or in the primary care setting. Such findings highlight a critical need to increase awareness of hand function in order to better identify impairments before they impede daily life and functional independence.

